# PDCD5 transfection increases cisplatin sensitivity and decreases invasion in hepatic cancer cells

**DOI:** 10.3892/ol.2014.2645

**Published:** 2014-10-29

**Authors:** GUI-LING FAN, YONG YAO, LI YAO, YUN LI

**Affiliations:** 1 Department of Gastroenterology, Shandong Qianfoshan Hospital, Shandong University, Jinan, Shandong 250014, P.R. China; 2 Department of General Surgery, The First People’s Hospital of Shanxian County, Heze, Shandong 274300, P.R. China; 3 Department of Dentistry, The Second Affiliated Hospital of Shandong University of Traditional Chinese Medicine, Jinan, Shandong 250001, P.R. China; 4 Department of Nursing, The First People’s Hospital of Shanxian County, Heze, Shandong 274300, P.R. China

**Keywords:** hepatocellular carcinoma, PDCD5, cisplatin, apoptosis, p53, epithelial-mesenchymal transition

## Abstract

Low expression levels of the programmed cell death 5 (PDCD5) gene have been reported in numerous human cancers, however, PDCD5 expression has not been investigated in hepatic cancer. The present study aims to investigate the biological behavior of PDCD5 overexpression in hepatocellular carcinoma (HCC) cells. The PDCD5 gene was stably transfected into the HepG2 HCC cell line (HepG2-PDCD5), and the expression levels of PDCD5 were examined by quantitative polymerase chain reaction and western blotting. An MTT assay was used to assess the cellular proliferating ability, and propidium iodide (PI) staining was used to evaluate the cell cycle by flow cytometry. The cells were incubated with 2 ng/ml transforming growth factor (TGF)-β for 7 days in order to induce invasion and epithelial-mesenchymal transition (EMT). Apoptosis was measured by Annexin V-fluorescein isothiocyanate and PI double labeling. A Boyden chamber invasion assay was carried out to detect tumor invasion. Western blotting was performed to detect the protein expression levels of PDCD5, insulin-like growth factor (IGF)-1 and the EMT marker, Snail. The results showed that the HepG2-PDCD5 cells exhibited slower proliferation rates and high G_2_/M cell numbers compared with those of the HepG2 and HepG2-Neo controls (P<0.05). The PDCD5 transfected cells showed higher sensitivity to cisplatin treatment than the HepG2-Neo cells, with a higher p53 protein expression level. PDCD5 overexpression can attenuate tumor invasion, EMT and the level of IGF-1 protein induced by TGF-β treatment. In conclusion, stable transfection of the PDCD5 gene can inhibit growth and induce cell cycle arrest in HepG2 cells, and its also notably improves the apoptosis-inducing effects of cisplatin, and reverses invasion and EMT induced by TGF-β. The use of PDCD5 is a novel strategy for improving the chemotherapeutic effects on HCC.

## Introduction

A number of studies have demonstrated that apoptosis is closely associated with the initiation, progression and recurrence of cancer (1–3). Therefore, it is important to manipulate apoptosis-regulating factors in the effective treatment of cancer patients. Human programmed cell death 5 (PDCD5), formerly designated as TF-1 cell apoptosis-related gene 19, was cloned from TF-1 cells during the apoptotic process induced by cytokine withdrawal. PDCD5 plays a significant role in cellular apoptosis, and its overexpression in TF-1, MGC-803 and HeLa cells facilitates apoptosis triggered by growth factor or serum withdrawal (4). PDCD5 is widely expressed in various tissues and its mRNA expression level is significantly higher in adult tissues than in fetal tissues. In cells undergoing apoptosis, the level of PDCD5 protein is significantly increased and is located in the nuclei preceding the externalization of phosphatidylserine and the fragmentation of chromosomal DNA (5).

The decreased expression of PDCD5 has been reported in various human tumors, including prostate (6), lung (7) and ovarian (8) cancer, gliomas (9) and leukemia (10). PDCD5 also enhances apoptosis by cooperating with cisplatin [cis-diamminedichloroplatinum(II)] in certain tumor cells, including those of colorectal cancer, gastric cancer and glioma (11–13).

Cisplatin is a common drug for cancer chemotherapy, however, with repeated exposure, tumor cells often become resistant to its effects. Hepatocellular carcinoma (HCC) is known to be resistant to various chemotherapeutics, including cisplatin. Studies have shown that this resistance is likely to be attributable to the cisplatin-induced upregulation of hTERT and the PI3K-dependent survivin pathway (14,15). Therefore, we hypothesize that the exogenous overexpression of PDCD5 may enhance apoptosis and reverse cisplatin resistance in HCC. In the present study, the biological behavior of HCC cells were analyzed *in vitro* by the stable transfection of the PDCD5 gene, and the effects on apoptosis induced by cisplatin and invasion by transforming growth factor (TGF)-β were investigated.

## Materials and methods

### Cell culture

The human HCC cell line, HepG2, was purchased from the Institute of Biochemistry and Cell Biology, Shanghai Institutes for Biological Sciences, Chinese Academy of Sciences (Shanghai, China). The cells were incubated in complete Dulbecco’s modified Eagle’s medium (DMEM; Invitrogen, Carlsbad, CA, USA) supplemented with 10% heat-inactivated fetal bovine serum (FBS; Sijichun Bioengineering Materials Inc., Hangzhou, Zhejiang, China), 100 U/ml penicillin and 100 μg/ml streptomycin, in a humidified incubator at 37°C with 5% CO_2_.

### Construction and transfection of PDCD5 plasmid

A PDCD5 full length cDNA sequence was obtained from GenBank (http://www.ncbi.nlm.nih.gov/genbank/; accession number, NM_004708.3). Total RNA was extracted using oligo (dT) from the human HCC HepG2 cells and was reverse transcribed as a template for reverse transcription polymerase chain reaction (RT-PCR). The primer sequences were as the follows: Sense, 5′-CGC GGA TCC CCG AGG GGC TGC GAG AGT GA-3′ and antisense, 5′-CGC GAA TTC CCT AGA CTT GTT CCG TTA AG-3′. PCR conditions of 40 cycles of 94°C for 30 sec, 60°C for 45 sec and 72°C for 30 sec followed by a final elongation step at 72°C for 10 min, were used. The PCR products of full-length PDCD5 cDNA were then ligated into the *Bam*HI/*Eco*RI sites of the eukaryotic expression vector, pcDNA3.1/Neo(+) (Invitrogen) using T4 DNA ligase at 16°C overnight, followed by transformation of competent *Escherichia coli* DH5α. DNA sequencing was used to identify a recombinant plasmid clone with the correct sequence, and this bacterial clone was amplified and purified in for eukaryote transfection. The HepG2 cells were transfected with pcDNA3.1-PDCD5 plasmid or pcDNA3.1-Neo plasmid (empty vector) [pcDNA3.1(+)] using Lipofectamine™ 2000 (Invitrogen, Carlsbad, CA, USA) according to the manufacturer’s instructions. RT-PCR and RT-quantitative (q)PCR were performed to detect PDCD5 mRNA expression 48 h after transfection. SuccessfulLY transfected HepG2 cells were then grown in complete medium for further G418 screening (400 μg/ml; Sigma-Aldrich, St. Louis, MO, USA). After four weeks, colonies were isolated and expanded into cell clones. The subclone cells expressing only Neo or Neo and PDCD5 genes were termed HepG2-Neo and HepG2-PDCD5, respectively.

### RT-PCR analysis

The levels of PDCD5 mRNA were first examined by RT-PCR and β-actin was used as an internal reference. Total RNA (5 μg) was isolated from the HepG2 cells 48 h after transfection and RT was performed to synthesize cDNA using random primers with Easyscript First-Strand cDNA Synthesis SuperMix (TransGen Biotech, Beijing, China) primed with oligo(dT_18_). The forward and reverse primers were synthesized by Sangon Biotech (Shanghai) Co., Ltd., (Beijing, China), and the sequences and expected sizes of the PCR products were as follows: PDCD5 forward, 5′-ACA GAT GGC AAG ATA TGG ACA-3′ and reverse, 5′-TCC TAG ACT TGT TCC GTT AAG-3′ (210bp); and β-actin forward, 5-CGG GAA ATC GTG CGT GAC ATT-3′ and reverse, 5′-CTA GAA GCA TTT GCG GTG GAC-3′ (510bp). The thermal procedure of PCR for PDCD5 and β-actin mRNA was performed at 94°C for 4 min for 1 cycle, then 94°C for 45 sec, 52°C for 45 sec and 72°C for 1 min for 30 cycles, and 72°C for 7 min for 1 cycle. PCR products were subjected to electrophoresis on 1.5% agarose gels containing ethidium bromide and then visualized under ultraviolet light.

### qPCR analysis

To quantify the results of the RT-PCR, PDCD5 mRNA expression levels were further analyzed by RT-qPCR analysis, which was performed by an RT-Cycler™ Real Time PCR Detection System (CapitalBio, Ltd., Beijing, China) with SYBR Green (Molecular Probes, Invitrogen). The following primers were used: Sense, 5′-ACA GAT GGC AAG ATA TGG ACA-3′ and anti-sense, 5′-TCC TAG ACT TGT TCC GTT AAG-3′ (199 bp) for PDCD5 and; sense, 5′-TTA GTT GCG TTA CAC CCT TTC-3′ and anti-sense, 5′-ACC TTC ACC GTT CCA GTT T-3′ (150 bp) for β-actin. Firstly, RNA was extracted and reverse transcribed to cDNA using Easyscript First-Strand cDNA Synthesis SuperMix (Beijing TransGen Biotech Co., Ltd., Beijing, China), then 1 μl cDNA was added in a 20-μl reaction mixture containing 0.5X SYBR Green, 1X TransStart Green qPCR Supermix (Transgen Biotech) and 0.5 μmol/l primer sets. The cycling conditions were as follows: 95°C for 5 min for 1 cycle, followed by 95°C for 45 sec, 57°C for 20 sec and 72°C for 20 sec for 40 cycles. The expression levels of PDCD5 were internally normalized to β-actin. The relative expression level of PDCD5 mRNA was calculated by the 2^−ΔΔCT^ method. Each experiment was performed in duplicate and repeated three times.

### Cell viability assay

The cell growth rate was determined by MTT assay (Sigma-Aldrich). Briefly, cells (100 μl) at the logarithmic growth phase were seeded at a 1×10^4^/ml density into 96-well culture plates. MTT solution (10 μl; 5 mg/ml) was added into each well and incubated at 37°C for 4 h. Following centrifugation at 1,409 × g for 10 min, the supernatant was discarded and 100 μl DMSO was added. When the remaining formazan pellet was dissolved completely, the absorbance values at a 570 nm wavelength were read on an ELISA plate reader (Bio-Rad, Hercules, CA, USA). The total procedure was repeated three times.

### Flow cytometry analysis of the cell cycle

HepG2 cells at the logarithmic growth phase were seeded in 6-well plates. After reaching 50% confluence, the adherent cells were cultured in serum-free medium for 24 h and then were cultured in DMEM supplemented with 10% FBS. After 48 h, the cells were digested and harvested with 250 μl trypsin. The cell pellet was obtained following centrifugation for 3 min at 4°C and 978 × g, and was re-suspended with 300 μl ice-cold PBS on ice, followed by resuspension in 70% ethanol at 4°C for 30 min. Finally, 1 ml propidium iodide (PI) staining solution (20 μg/ml PI, 0.1% Triton X-100, 2 mM EDTA and 8 μg/ml DNase-free RNase) was added to the samples, and then the associated data were analyzed on a FACScan (Becton-Dickinson, San Francisco, CA, USA). Results were acquired from 10,000 cells.

### Flow cytometric analysis of apoptosis

Next, an Annexin V-fluorescein isothiocyanate (FITC) apoptosis detection kit (BD Biosciences Clontech, California) was used to identify the translocation of phosphatidylserine. The HepG2 cells were cultured with 5 μg/ml cisplatin. After 24 h, 2×10^5^ cells in each well were harvested. Subsequent to being washed with PBS buffer, the cell pellet was incubated with 2.5 μl Annexin V and 5 μl PI (final concentration, 10 μg/ml) in 100 μl 1X binding buffer for 15 min in the dark. Apoptosis was determined by flow cytometry and analyzed using CellQuest and Modfit software (Becton-Dickinson). At least 10,000 events were analyzed for each sample.

### Cell migration assay

The Boyden chamber invasion assay was performed to evaluate the *in vitro* migration of the HepG2-Neo and HepG2-PDCD5 cells in a 24-well tissue culture plate with a Transwell filter membrane. The lower side of the filters was coated with type I collagen (0.5 mg/ml). The cells were seeded in the upper part of the Transwell plate at a density of 5×10^5^/ml, with 100 μl cell suspension in each well. After 24 h, the cells on the upper surface of the filter were removed, and the remaining cells were fixed with methanol and stained with hematoxylin and eosin (Sigma-Aldrich). Cell counting was performed under light microscopy (x200 magnification) and the cells that had migrated to the lower chamber were regarded as migrated cells. Each sample was assayed in triplicate and repeated twice

### Western blot analysis

Proteins from the HepG2 cells were extracted and their concentrations were determined by bicinchoninic acid protein concentration assay kit (Beijing Biosea Biotechnology, Co., Ltd., Beijing, China). The cell lysates (50 μg) were resolved on 15% SDS-polyacrylamide gels, electrophoretically transferred to polyvinylidene difluoride membranes and then incubated with primary monoclonal mouse antibodies against PDCD5, phospho-p53, Snail or insulin-like growth factor (IGF)-1 (Santa Cruz Biotechnology, Inc., Santa Cruz, CA, USA). The horseradish peroxidase-conjugated rabbit anti-mouse secondary antibody was used at 1:1,000 dilutions for 2 h at room temperature. Blots were visualized using the chemiluminescence method. β-actin was used as an internal control.

### Statistical analysis

All quantitative data are expressed as the mean ± standard deviation. The statistical analysis was performed by commercially available SPSS 14.0 software (SPSS, Inc., Chicago, IL, USA). Student’s t-test (unpaired, two-tailed) was performed to compare the means between two groups. The means of the different groups were compared using a one-way analysis of variance. P<0.05 was used to indicate a statistically significant difference.

## Results

### Construction of eukaryotic expression vector of pcDNA3.1/Neo(+)-PDCD5

**Figure 1:**
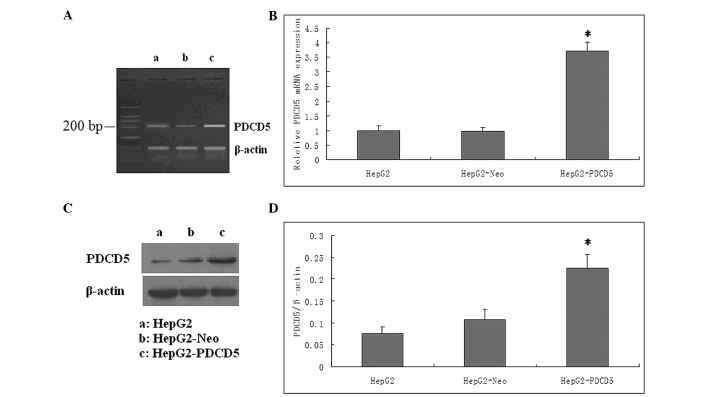
Validation for the efficacy of PDCD5 overexpression at the mRNA and protein levels. (A) The PDCD5 mRNA expression level was markedly increased by pcDNA3.1-PDCD5 plasmid transfection, as shown by the reverse transcription polymerase chain reaction (RT-PCR) products in the agarose gel. a, HepG2 cells; b, HepG2-Neo cells; c, HepG2-PDCD5 cells (B) Expression of PDCD5 mRNA was detected by RT-quantitative PCR analysis. The level of PDCD5 mRNA following transfection was increased by three-fold (P<0.05). The control plasmid had no effect on the PDCD5 mRNA expression level in the HepG2 cells. (C) PDCD5 protein expression was detected by western blot analysis. PDCD5 protein expression was higher in the cells transfected with PDCD5 than in the wild-type cells or cells transfected with control plasmid. β-actin served as a loading control. One representative figure is shown from three independent experiments. a, HepG2 cells; b, HepG2-Neo cells; c, HepG2-PDCD5 cells (D) Relative expression of PDCD5 of the three groups. The Y axis indicates the gray value of PDCD5 normalized to that of β-actin. Data are expressed as the mean ± standard deviation. A two-tailed, unpaired t-test was performed. ^*^P<0.05 vs. HepG2 group (n=6). PDCD5, programmed cell death 5; HepG2-Neo, HepG2 cells transfected with control plamid; HepG2-PDCD5, HepG2 cells transfected with PDCD5 plamid.

Following the DNA sequencing analysis for the validation of recombinant pcDNA3.1/Neo(+)-PDCD5 (data not shown), G418 screening was performed to select for cells with successful transfection. All untransfected HepG2 cells were dead following G418 (400 μg/ml) selection. The pcDNA3.1/Neo(+)-PDCD5 transfected cells were cultivated with G418 for four weeks, until viable clones could be observed. The clones were selected for further amplification. The RT-PCR, RT-qPCR and western blot analyses were performed to measure the mRNA and protein expression levels of PDCD5 in the HepG2, HepG2-Neo and HepG2-PDCD5 cells. The mRNA and protein levels of PDCD5 in the HepG2-PDCD5 cells were significantly higher than those in the HepG2 and HepG2-Neo cells (P<0.05). No significant difference was found in PDCD5 expression between the HepG2 and HepG2-Neo cells (P>0.05) (Fig. 1).

### Changes of the biological behavior of the tumor by the stable transfection of PDCD5

**Figure 2:**
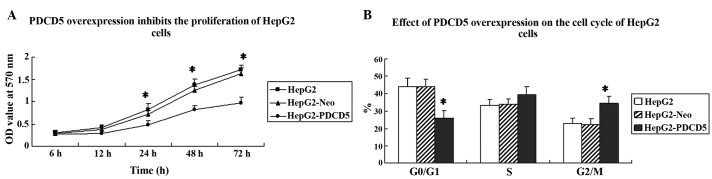
PDCD5 overexpression changes tumor biology morphology in HepG2 cells. (A) Growth curves of the cells based on data from the MTT assay in normal HepG2 cells, and HepG2 cells transfected with control plamid (HepG2-Neo) or PDCD5 plamid (HepG2-PDCD5) at 6, 12, 24, 48 and 72 h. From 24 h, the viability of the HepG2 cells stably expressing PDCD5 was significantly lower than that of the HepG2 cells (P<0.05). (B) PDCD5 overexpression induced cell cycle arrest at the G_2_/M phase in the HepG2 cells. The HepG2, HepG2-Neo and HepG2-PDCD5 cells were cultured in serum-free medium for 24 h and then were cultured in Dulbecco’s modified Eagle’s medioum supplemented with 10% fetal bovine serum for a further 48 h. Propidium iodide (20 μg/ml) staining was performed to determine the percentages of cells in the G_0_/G_1_, S and G_2_/M phases. ^*^P<0.05 vs. HepG2 group (n=6). PDCD5, programmed cell death 5.

To analyze the effect of PDCD5 overexpression on cancer cell growth, an MTT assay and flow cytometry were performed to assess cell proliferation and the cell cycle. Cell proliferation was significantly slower in the HepG2-PDCD5 cells compared with the HepG2 and HepG2-Neo cells (Fig. 2A). Next, an analysis of the cell cycle was performed on the HepG2, HepG2-Neo and HepG2-PDCD5 cells. The percentage of cells in the G_2_/M phase was significantly higher in the HepG2-PDCD5 cells compared with the HepG2 and HepG2-Neo cells (P<0.05) (Fig. 2B). This indicated that PDCD5 overexpression induces G_2_/M cell cycle arrest in HepG2 cells.

### PDCD5 protein enhances the sensitivity of HepG2 cells to cisplatin in vitro

**Figure 3:**
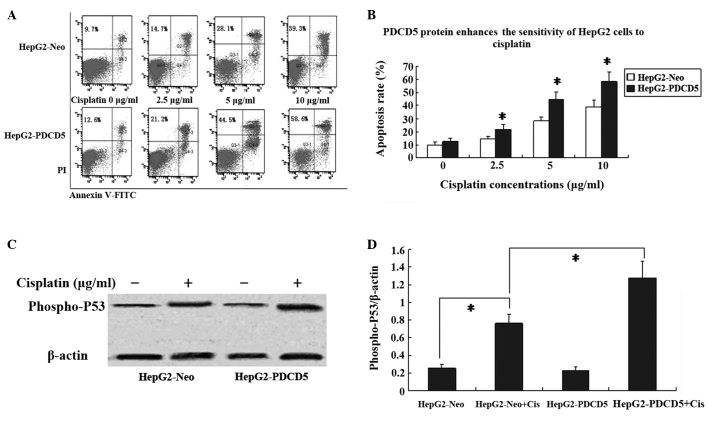
PDCD5 protein enhances the sensitivity of HepG2 cells to cisplatin. (A) HepG2-Neo and HepG2-PDCD5 cells were treated with various concentrations of cisplatin (0, 2.5, 5, 10 μg/ml) for 24 h. Apoptosis was determined through Annexin V-fluorescein isothiocyanate (FITC) and propidium iodide (PI) double staining using flow cytometry. PDCD5 overexpression alone did not increase the apoptotic rate in the HepG2 cells, but it did increase the apoptotic rate in the HepG2 cells treated with cisplatin. Representative images from three experiments are shown. (B) Apoptotic rates in the HepG2-Neo and HepG2-PDCD5 cells treated with various concentrations of cisplatin. Data are expressed as the mean ± standard deviation and were compared using a two-tailed, unpaired t-test. ^*^P<0.05 vs. HepG2-Neo group (n=3). (C) Following cisplatin treatment, PDCD5 overexpression increased the phospho-p53 protein level in the HepG2 cells following cisplatin treatment. The HepG2-Neo and HepG2-PDCD5 cells were treated with or without cisplatin (5 μg/ml) for 24 h. The level of phospho-p53 protein was then determined by western blotting. β-actin served as a loading control. One representative figure is shown from three independent experiments. (D) Relative expression of phospho-p53 protein of the four groups. The Y axis indicates the gray value of phospho-p53 protein normalized to that of β-actin. Data are expressed as the mean ± standard deviation. A two-tailed, unpaired t-test was performed. ^*^P<0.05 (n=3). HepG2-Neo, HepG2 cells transfected with control plamid; HepG2-PDCD5, HepG2 cells transfected with PDCD5 plamid.

The HepG2-Neo and HepG2-PDCD5 cells were treated with varying concentrations of cisplatin and the chemotherapeutic sensitivity was assessed by Annexin V-FITC and PI double staining. Following treatment with 2.5, 5 and 10 μg/ml cisplatin for 24 h, the number of HepG2-Neo and HepG2-PDCD5 apoptotic cells were increased in a dose-dependent manner (Fig. 3A and B). In the cells without cisplatin treatment, no difference was found in the apoptotic rate between the HepG2-Neo and HepG2-PDCD5 cells.

PDCD5 interacts with p53 proteins in a variety of cancer cells. To assess the effect of PDCD5 on p53 protein, the level of phospho-p53 protein was measured by western blot analysis. Phospho-p53 protein expression was increased following 24 h of treatment with cisplatin in the HepG2-Neo and HepG2-PDCD5 cells. Moreover, the HepG2-PDCD5 cells showed higher phospho-p53 protein levels compared with the HepG2-Neo cells following cisplatin treatment (Fig. 3C and D). No difference was found in the p53 protein expression level between the HepG2-Neo and HepG2-PDCD5 cells without cisplatin treatment.

### PDCD5 overexpression reduces invasion and epithelial-mesenchymal transition (EMT) induced by TGF-β

**Figure 4:**
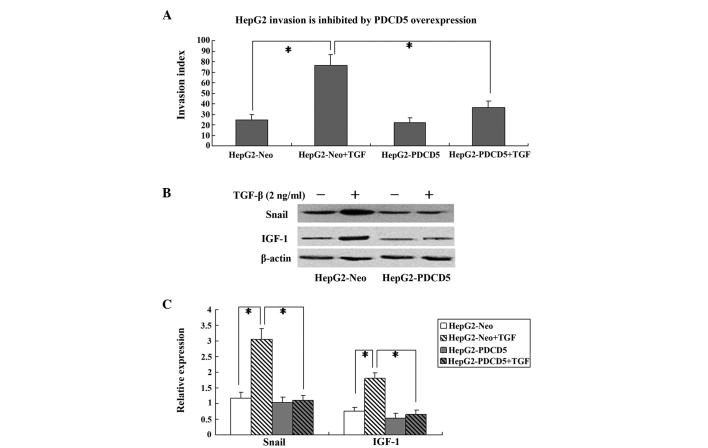
PDCD5 overexpression reduces invasion and EMT induced by TGF-β. The HepG2-Neo and HepG2-PDCD5 cells were incubated with TGF-β (2 ng/ml) for 24 h. (A) A Boyden Chamber assay was performed to detect the inhibitory effect of PDCD5 on cell invasion induced by TGF-β. (B) PDCD5 inhibited the Snail and IGF-1 protein expression induced by TGF-β. Western blotting was performed to detect Snail and IGF-1 protein levels in the HepG2 cells treated with TGF-β (2 ng/ml) or without for 24 h. β-actin served as a loading control. One representative figure is shown from three independent experiments. (C) Relative expression of Snail and IGF-1 protein of the four groups. The Y axis indicates the gray value of Snail or IGF-1 normalized to that of β-actin. Data are expressed as the mean ± standard deviation and a two-tailed, unpaired t-test was performed. ^*^P<0.05 (n=3). HepG2-Neo, HepG2 cells transfected with control plamid; HepG2-PDCD5, HepG2 cells transfected with PDCD5 plamid; TGF-β, transforming growth factor-β; IGF-1, insulin-like growth factor 1.

The HepG2-Neo and HepG2-PDCD5 cells were incubated with TGF-β (2 ng/ml) for 24 h to induce invasion and EMT. The Boyden chamber invasion assay showed that PDCD5 transfection exhibited no effect on the invasion index in the cells without TGF-β treatment. However, in the TGF-β-treated HepG2 cells, PDCD5 transfection significantly decreased the invasion index compared with the cells transfected with the control plasmid (P<0.05) (Fig. 4A).

The expression of the EMT marker, Snail, was determined by western blotting. In the cells transfected with the HepG2-Neo control plasmid, Snail protein was significantly increased by TGF-β treatment. However, PDCD5 transfection significantly decreased Snail protein expression in the HepG2 cells treated with TGF-β (P<0.05) (Fig. 4B and C).

IGF-1 protein expression was also measured in the HepG2 cells by western blotting. TGF-β treatment significantly increased the IGF-1 protein expression level in the HepG2-Neo cells, which was attenuated in the HepG2-PDCD5 cells (Fig. 4B and C). This indicates that PDCD5 may downregulate IGF-1 protein expression in TGF-β-treated HepG2 cells.

## Discussion

In the present study, the PDCD5 gene was stably transfected into a human HCC cell line to induce its overexpression. It was demonstrated that the transfection of PDCD5 into HepG2 cells could change the biological behaviors of tumors, such as the cellular proliferation, cell cycle progression, cisplatin sensitivity, tumor invasion and EMT. The growth of the HepG2-PDCD5 cells was slower than that of the HepG2 and HepG2-Neo cells. A higher percentage of HepG2-PDCD5 cells were in the G_2_/M phase compared with the HepG2 and HepG2-Neo cells. The PDCD5-transfected cells showed higher sensitivity to cisplatin treatment compared with the HepG2-Neo cells, with higher p53 protein expression. PDCD5 overexpression can attenuate tumor invasion, EMT and IGF-1 protein induced by TGF-β treatment.

In the present study, the HepG2-PDCD5 cells had decreased levels of proliferation compared with the HepG2 and HepG2-Neo cells, as demonstrated by the presence of less viable cells. This indicated that PDCD5 may participate in the pathogenesis of tumors, and will be associated with various clinicopathological factors in HCC patients. The decreased expression level of PDCD5 has been observed in various human tumors, and is correlated with high-grade astrocytic gliomas (9), a higher Gleason grade in prostate cancer (6) and an advanced International Federation of Gynecologists and Obstetricians stage and poorer survival in epithelial ovarian carcinomas (8). To the best of our knowledge, no study has been reported on the correlation between PDCD5 expression and the clinicopathological factors in HCC patients; this requires further investigation.

The present study found that the stable transfection of PDCD5 could also induce cell cycle arrest, as demonstrated by the higher percentage of HepG2-PDCD5 cells in the G_2_/M phase compared with the HepG2 and HepG2-Neo cells. This suggests that the decreased number of viable HepG2-PDCD5 cells is partly caused by the inhibition of cell cycle progression. G_2_/M is an important cell cycle checkpoint prior to cells entering the mitotic phase. In the present study, G_2_/M arrest following PDCD5 transfection depended on functional p53, since in cells with inactive p53, G_2_/M arrest did not occur (16,17). This indicates that in HepG2 cells, p53 protein is intact and cisplatin resistance could be reversed (18). Therefore, the present study evaluated whether PDCD5 overexpression enhances the sensitivity of HepG2 cells to cisplatin *in vitro*.

Cisplatin is a second-generation platinum-based chemotherapeutic drug for the clinical treatment of a variety of tumors. Cisplatin functions through restraining DNA replication, leading to cell cycle arrest and apoptosis. The long-term use of cisplatin results the development of drug resistance in numerous patients with HCC, leading to disease recurrence (19). Therefore, increasing the sensitivity of HCC cells to cisplatin may aid in overcoming drug resistance and tumor recurrence. The present results demonstrated that HepG2 cells with stable transfection of PDCD5 exhibited an enhanced sensitivity to cisplatin (5 μg/ml) exposure, as demonstrated by the higher apoptosis rates compared with the HepG2-Neo cells. The aforementioned results showed that PDCD5 can induce G_2_/M phase cell cycle arrest in HepG2 cells, and indicates functional p53 protein within the cells. Therefore, the study investigated whether p53 is able to participate in the enhanced sensitivity to cisplatin caused by PDCD5. The results showed that higher phospho-p53 protein levels were observed in the HepG2-PDCD5 cells compared with the HepG2-Neo cells following cisplatin treatment, and suggests that elevated phospho-p53 protein levels may mediate enhanced apoptosis by PDCD5 transfection following cisplatin treatment. In fact, PDCD5 can directly bind with p53 protein (20), and co-localization of p53 and PDCD5 protein has been found in the synergistic therapeutic effect of PDCD5 with cisplatin (12). The present results were further supported by a recent study that showed that the knockdown of PDCD5 by RNA interference decreased the level of p53 phosphorylation (21). This indicates that PDCD5 may function as a co-activator of p53 upon DNA damage, such as that caused by cisplatin treatment.

The present study also found that PDCD5 overexpression could reduce invasion and EMT induced by TGF-β. Tumor invasion is a complex and multi-step process that involves alteration of the cell adhesion to extracellular matrix proteins. TGF-β has been shown to promote vascular invasion in HCC (22). The present results showed that the pro-invasive effect of TGF-β was reversed by PDCD5 overexpression. There are few studies on the correlation between PDCD5 and tumor invasion. However, a correlation was indicated in rheumatoid arthritis, an inflammatory disease. The PDCD5 levels in the plasma and synovial fluid of patient with rheumatoid arthritis were shown to be inversely associated with two inflammatory cytokines, tumor necrosis factor (TNF)-α and interleukin (IL)-17 (23,24). TNF-α-mediated nuclear factor-κB expression promotes the invasion of HCC cells, and its downregulation mediates the anti-invasive effect of a number of drugs (25). IL-17 can also promote the invasion and metastasis of HCC cells (26).

EMT is one vital step in epithelial cells, characterized by loss of cell adhesion and acquisition of malignant phenotype, including capabilities of cell motility, migration, invasion and metastasis to a new location (27). The present results showed that following TGF-β treatment, compared with the HepG2-Neo cells, the HepG2-PDCD5 cells showed decreased expression levels of Snail, which is an EMT marker protein. This means that PDCD5 has an inhibitory effect on EMT in HCC cells, and provided a novel anti-tumor strategy for treating HCC.

The present study found that TGF-β treatment significantly increased IGF-1 protein expression in the HepG2-Neo cells, which was attenuated in the HepG2-PDCD5 cells, indicating negative regulation on IGF-1 by PDCD5. IGF-1 is mainly secreted by the liver as a result of stimulation by growth hormone. IGF-1 plays roles in the promotion of cell proliferation and the inhibition of apoptosis. In human prostate cancer cells, IGF-1 upregulates ZEB1 and drives EMT (28). The present results validated the effect of IGF-1 on EMT in HCC cells. Recently, a study showed negative correlations between IGF-1 and PDCD5 at the mRNA and protein levels (29). The study inferred that IGF-1 may downregulate the expression of PDCD5. However, the present results indicate that IGF-1 may be downregulated by PDCD5. This disparity requires further investigation.

In conclusion, stable transfection of the PDCD5 gene can inhibit the growth and induce cell cycle arrest in the G_2_/M phase in HepG2 cells. PDCD5 transfection can also enhance the sensitivity to cisplatin and p53 phosphorylation, and reverse invasion and EMT induced by TGF-β. PDCD5 represents a novel drug target and therapeutic strategy for the improved chemotherapeutic treatment of HCC.
